# Analysis of incidence and risk factors of the multidrug resistant gastrointestinal tract infection in children and adolescents undergoing allogeneic and autologous hematopoietic cell transplantation: a nationwide study

**DOI:** 10.1007/s00277-021-04681-y

**Published:** 2021-10-21

**Authors:** Małgorzata Salamonowicz-Bodzioch, Jowita Frączkiewicz, Krzysztof Czyżewski, Olga Zając-Spychała, Ewa Gorczyńska, Grażyna Wróbel, Bernarda Kazanowska, Dorota Sęga-Pondel, Jadwiga Węcławek-Tompol, Marek Ussowicz, Krzysztof Kałwak, Mariusz Wysocki, Magdalena Dziedzic, Jacek Wachowiak, Agnieszka Zaucha-Prażmo, Jerzy Kowalczyk, Jolanta Goździk, Jan Styczyński

**Affiliations:** 1 Department of Pediatric Stem Cell Transplantation, Hematology and Oncology, Wroclaw Medical University, ul. Borowska 213, 50-556, Wroclaw, Poland; 2 Department of Pediatric Hematology and Oncology, Collegium Medicum, Nicolaus Copernicus University Torun, Bydgoszcz, Poland; 3 Department of Pediatric Oncology, Hematology and Transplantology, University of Medical Sciences, Poznan, Poland; 4 Department of Pediatric Hematology, Oncology and Stem Cell Transplantation, Medical University of Lublin, Lublin, Poland; 5 Stem Cell Transplant Center, Department of Clinical Immunology and Transplantology, University Children’s Hospital, Jagiellonian University Collegium Medicum, Krakow, Poland

**Keywords:** Gastrointestinal tract infection, *Clostridium difficile*, Gram-negative multidrug-resistant bacteria, Malignant diseases, Pediatric hematology and oncology, Hematopoietic stem cell transplantation, Autologous transplantation

## Abstract

The aim of this multi-center study was to evaluate the incidence, clinical course, and risk factors for bacterial multidrug-resistant (MDR) gastrointestinal tract infections (GTI) among children undergoing allogeneic and autologous hematopoietic cell transplantation. A total number of 175 pediatric patients (aged 1–18 years), transplanted between January 2018 and December 2019, who were tested for bacterial colonization/infection were enrolled into this multi-center analysis. Episodes of MDR GTI occurred in 77/175 (44%) patients. In multivariate analysis for higher GTI incidence, the following factors were significant: matched-unrelated donor (MUD) transplantation, HLA mismatch, presence of graft-versus-host disease (GVHD), and gut GVHD. The most common GTI were *Clostridium difficile* (CDI), *multidrug-resistant Enterobacteriaceae* (*Klebsiella pneumoniae*, *Escherichia coli* extended-spectrum β-lactamase), and *Enterococcus* HLAR (high-level aminoglycoside-resistant). No MDR GTI–attributed deaths were reported. MDR GTI is a frequent complication after HCT among children, causes prolonged hospitalization, but rarely contributes to death. We identified risk factors of MDR GTI development in children, with focus on GVHD and unrelated donor and HLA mismatch. We conclude that the presence of Clostridiales plays an important anti-inflammatory homeostatic role and decreases incidence of GVHD or alleviate its course.

**Supplementary Information:**

The online version contains supplementary material available at 10.1007/s00277-021-04681-y.

## Introduction

Hematopoietic stem cell transplant recipients are at very high risk of bacteremia which can occur early after transplantation. There are two major insults to their innate immune system. Firstly, they have prolonged neutropenia and lack of phagocytes. Secondly, conditioning regimen leads to severe gastrointestinal (GI) mucositis, causing damage of their mucosal barrier. These two factors establish a high-risk setting for bacteremia caused by enteric organisms and other severe complications from these infections [1]. Functional and kinetic immune recovery in the allogeneic HCT recipient are influenced by donor’s factor (age, gender, immune function), graft (graft source, HLA matching), treatment (type of conditioning regimen, total body irradiation), recipient’s factor (age, presence of comorbidities, underlying disease previous therapy, organ dysfunction, changes in microbiome), and HCT complications (presence of GVHD, use of supportive care, or other medications). Defects and delays in immune reconstitution strongly associate with decreased overall survival and increased transplant-related morbidity and mortality [2].

One of the major challenges among HCT recipients are multidrug-resistant (MDR) bacterial pathogens: MDR *Enterobacteriaceae*, including extended-spectrum β-lactamase (ESBL)–producing and carbapenem-resistant *Enterobacteriaceae* (CRE), *Pseudomonas aeruginosa* metallo-beta-lactamase producing (MBL), *AMP*c strains, high-level aminoglycoside-resistant *Enterococcus* (HLAR), and vancomycin-resistant *Enterococcus* (VRE). These bacteria are very common causes of infections, associated with the high mortality rates. Carbapenems remain the “gold standard” treatment for serious infections due to ESBL-producing *Enterobacteriaceae* in HSCT recipients. Administration of β-lactam agents as an extended infusion is associated with better outcomes in patients with severe infections caused by *P. aeruginosa*. Older medications used for the treatment of CRE and MDR *P. aeruginosa* infections, such as polymyxins and aminoglycosides, have major limitations. On the other hand, new agents, such as ceftazidime-avibactam and ceftolozane-tazobactam, have a great potential for the treatment of *Klebsiella pneumoniae* carbapemenase-producing CRE and MDR *P. aeruginosa*, respectively, but more clinical data are needed to better evaluate their efficacy [1, 3].

The impact of the gut microbiota on anticancer therapy, including allo-HCT, receives increased attention nowadays. Recent studies in mice and humans suggest important relationships between the gut microbiota and outcome of allo-HCT. Currently several clinical trials on the role of microbiota in allo-HCT recipients are ongoing [3]. Associations between certain bacteria and allogeneic transplant outcomes have been found in many studies which have shown that the commensal microorganisms residing in the lumen of the intestines play an important role in the pathophysiology of GVHD [4, 5]. The aim of this multi-center study was to determine the incidence, risk factors, clinical course, and outcome of MDR GTI in pediatric patients after allogeneic and autologous HCT. We hypothesized there are transplant-related risk factors contributing to GI tract infections with MDR bacteria.

## Materials and methods

### Design of the study

Consecutive pediatric patients (aged 1–18 years), transplanted between January 2018 and December 2019, were enrolled into the study of monitoring for MDR GTI infections before and after allo- and auto-HCT and followed up for a period of minimum of 6 months or death, whichever occurred first. Children were transplanted in all 5 pediatric transplant centers in Poland (Poznan, Wroclaw, Bydgoszcz, Krakow, and Lublin). The centers did not differ between each other with respect to type of conditioning regimens, approach to GTI management, and hematological standard of care. One hundred seventy-five pediatric subjects, who underwent allogeneic or autologous transplantation and have been checked for MDR GTI, were enrolled into the study. Patients were excluded if they have been PCR-positive for CMV, ADV, EBV, and BKV viruses at the time of GTI testing and have been actively treated due to another bacterial, fungal, or parasitic infection. A consort diagram for the inclusion process is presented in the Supplementary materials.

Patients were divided into 2 groups: GTI1 and GTI2. GTI1, control group, included patients GTI-microbiologically MDR negative, without clinical symptoms, and GTI2 patients, with symptoms of gastrointestinal infection (fever, diarrhea, abdominal pain, vomiting, changes in abdominal ultrasound) and positive stool culture for MDR bacteria. Treatment with broad-spectrum antibiotics and supportive therapy (analgesics, hydration, antiemetic drugs, anti-diarrheal drugs) was administered to patients in this group. We excluded all other possible causes of vomiting, diarrhea, fever, and gastric pain before we classified patient as GTI positive.

Upon inclusion, a written informed consent was obtained from all patients above 7 years old and parents of participants (< 7 years old). The inclusions were made by a transplant physician. The dataset including age, gender, clinical findings, laboratory results, and predisposing factors was obtained. The inclusion criteria in the control group were solely GTI negative result for MDR (stool culture) and lack of symptoms in HCT subjects. The study was approved by the Bioethical Committee in Bydgoszcz (no. KB 499/2014).

### Conditioning regimens

#### *Allogenic setting*

Patients were treated with myeloablative conditioning (MAC), reduced-toxicity regimens (RTC), and reduced intensity protocol (RIC). Reduced-toxicity conditioning (RTC) consisted mainly of treosulfan (36–42 mg/m^2^), fludarabine (160 mg/m^2^), and with thiotepa (10 mg/kg), melphalan (140 mg/m^2^), or cyclophosphamide (120 mg/kg). In RIC regimen, fludarabine with either melphalan or low, non-myeloablative doses of busulfan (2 mg/kg) was used. MAC based either on myeloablative doses (12.8–19.6 mg/kg) of busulfan or on total body irradiation (TBI) at a median dose of 12 Gy [6]. Type of donor, graft source, reconstitution day, and conditioning regimen are presented in Table 1.

**Table 1:** Characteristics of patients

Total number of patients	175
Sex	
Male	108 (61.7%)
Female	67 (38.3%)
Age	
> 5 years	106 (60.6%)
< 5 years	69 (39.4%)
Diagnosis	
Acute lymphoblastic leukemia (ALL)	43 (24.6%)
Acute myeloblastic leukemia (AML)	18 (10.3%)
Severe aplastic anemia (SAA)	18 (10.3%)
Myelodysplastic syndrome (MDS)	6 (3.4%)
Hemophagocytic Lymphohistiocytosis (HLH)	2 (1.1%)
Non-Hodgkin lymphoma (NHL)	3 (1.7%)
Juvenile myelomonocytic leukemia (JMML)	3 (1.7%)
Primary immunodeficiency (PID)	14 (8.0%)
Neuroblastoma(NBL)	22 (12.6%)
Ewing sarcoma (ES)	15 (8.6%)
Hodgkin lymphoma(HD)	9 (5.1%)
Germ cell tumor	4 (2.3%)
Other	18 (10.3%)
Donor	
Matched unrelated donor (MUD)	73 (41.7%)
Matched sibling donor (MSD)	32 (18.3%)
Mismatched unrelated donor (MMUD)	7 (4.0%)
Haplo	5 (2.9%)
AUTO	58 (33.1%)
Neutrophil recovery	
Day < 15 after HCT	83 (47.4%)
Day ≥ 15 after HCT	92 (52.6%)

Legend. TBI-VP, total body irradiation-etoposide; TREO-FLU-TT, treosulfan-fludarabine-thiotepa; BU-FLU-TT, busulfan-fludarabine-thiotepa; BU-CY-MEL, busulfan-cyclophosphamide-melphalan; CY-FLU, cyclophosphamide-fludarabine; TREO-MEL, treosulfan-melphalan; BU-MEL, busulfan-melphalan; TREO-FLU, treosulfan-fludarabine; ALL, acute lymphoblastic leukemia; AML, acute myeloid leukemia; SAA, severe aplastic anemia; FA, Fanconi anemia; MDS, myelodysplastic syndromes; HLH, hemophagocytic lymphohistiocytosis; JMML, juvenile myelomonocytic leukemia; NHL, non-Hodgkin lymphoma; HD, Hodgkin lymphoma; NBL, neuroblastoma; ES, Ewing sarcoma; GERM, germ cell tumor; PID, primary immunodeficiencies; MSD, matched sibling donor; CB, cord blood; MUD, matched unrelated donor; MMUD, mismatched unrelated donor PB, peripheral blood; BM, bone marrow

As a standard GVHD prophylaxis, all patients, since 1 day before HCT, received intravenous cyclosporine (CsA) in a dose of 1.5 mg/kg in 2-h infusion twice a day. Furthermore, dosage of CsA was adjusted depending on the CsA level (target level: 100–200 μg/L), checked twice a week, and then switched into oral suspension (in stable patients who were able to accept oral intake). According to standard protocols, in patients with no signs of GVHD, CsA administration was continued until the day + 120, prefaced by slow tapering. The second prophylactic drug, methotrexate (MTX), was administered three times, in a standard dose of 10 mg/m^2^ on days 1, 3, and 6 after HCT. All patients transplanted from alternative donors or treated for severe aplastic anemia (SAA) were given in vivo T cell depletion by either rabbit anti-thymocyte globulin (ATG) (ATG-Fresenius/Grafalon® at a median dose 45 mg/kg or Thymoglobuline® at median dose 7.5 mg/kg) or Campath-1H at median dose 1 mg/kg [6].

#### *Autologous setting*

Solid tumor protocols consisted of various combinations of treosulfan, busulfan, cyclophosphamide, melphalan, and fludarabine. In majority of neuroblastoma (NBL) cases, busulfan-melphalan (BU-MEL)-based high-dose chemotherapy (HDC) was administered. Patients suffering from Ewing sarcoma (ES) were conditioned mainly with treosulfan-melphalan (TREO-MEL) and busulfan-melphalan (BU-MEL) regimens.

#### *Prophylaxis of infections*

All participating patients were followed starting from day of transplantation up to at least 100 days post transplantation. All were hospitalized in single-bed rooms.

In case of negative stool culture, any particular prophylaxis was not used (control group, GTI1). In case of colonization with Gram-negative or Gram-positive MDR bacteria, antimicrobial prophylaxis consisted of oral colistin or rifaximin was used from the beginning of the conditioning regimen. In case of positive binary toxin or B, or A *Clostridium difficile* toxin before HDC, treatment with oral metronidazole (3 × 10 mg/kg) was used for 10–14 days. In case of busulfan-containing regimens, oral vancomycin (4 × 10 mg/kg) was administered. In case of severe CDI, both metronidazole and vancomycin were administered [7]. First-line intravenous antibiotic empiric therapy usually included a broad-spectrum beta-lactam (piperacillin with tazobactam, or cefepime) and aminoglycoside. Blood cultures were taken at the start of fever and whenever antibiotics were exchanged. In the case of MDR Gram-negative rods, carbapenems were used as a first choice.

Intravenous or oral acyclovir and oral posaconazole were started at the onset of conditioning regimen. Prophylactic trimethoprim/sulfamethoxazole was given orally to all patients before and after HCT until at least 1 month after the end of immunosuppression. Antifungal prophylaxis with posaconazole was continued until the end of immunosuppressive therapy. First-line intravenous antibiotic empiric therapy usually included aminoglycoside and a broad-spectrum beta-lactam [6].

#### Diagnosis of GTI

Gastrointestinal tract infection was established in patients with typical symptoms (abdominal pain, diarrhea, vomiting, fever, abnormalities in ultrasound) and presence of positive stool culture for MDR bacteria. CDI was diagnosed in patients having diarrhea (more than three semiliquid or liquid stools within 24 h) without evidence of another cause (rotavirus, adenovirus) and a positive stool test result for the presence of toxigenic *C. difficile* (as detected by enzyme immunoassay, polymerase chain reaction or microbial culture). Stool specimens were analyzed every time having diarrhea or/and once a week (HCT) without symptoms as a routine control. Additionally all asymptomatic GTI patients were screened weekly for the presence of other viruses (ADV, CMV, Epstein-Barr virus, rotavirus), and multi-resistant bacteria, as a part of routine monitoring [6, 7].

Differentiation between GTI and GVHD was based on clinical experience, infectious laboratory, and radiological checking. In patients suspected to develop gut GVHD, colonoscopy/or/and gastroscopy were performed.

Colonization with Gram-positive/Gram-negative MDR bacteria was defined as having positive stool culture without symptoms at a moment of admission to the transplant ward before start of conditioning regimen.

#### Treatment of GTI

In case of infection, broad-spectrum antibiotics were used. The empiric therapy consisted of broad-spectrum beta-lactams and aminoglycosides. In case of extended-spectrum β-lactamase and carbapenemase producers (ESBL), carbapenems were used. In culture positive for vancomycin-resistant *Enterococci*, patients were treated with teicoplanin or linezolid.

In case of positive binary toxin or B, or A *Clostridium difficile* toxin and symptoms typical for CDI, treatment with oral metronidazole (3 × 10 mg/kg) was carried up for 10–14 days. In case of severe CDI, metronidazole and vancomycin were administered intravenously [7].

### Statistical analysis

To compare differences between groups, the chi-square test or Fisher exact test was used for categorical variables and the Mann–Whitney *U* test for continuous variables. Odds ratio (OR) and 95% confidence intervals (95% CI) are shown. The cumulative incidences of GTI infection were assessed using competing risk analysis and Gray’s test. A multivariate logistic regression using the stepwise model selection method was used to evaluate potential risk factors that might influence donor outcome variables. The following risk factors were analyzed: age, conditioning regimen, type of donor, HLA match, cell source, neutrophil engraftment, presence of GVHD, and *p* < 0.05 were regarded as significant [6].

## Results

### Demographics

A total number of 175 (117 allo-HCT and 58 auto-HCT) patients (GTI1 + GTI2), including 67 girls and 108 boys, were included to the study, with a median age 7.1 (range 0.3–23.5 years). Children were transplanted due to acute lymphoblastic leukemia (ALL; *n* = 43), acute myeloblastic leukemia (AML; *n* = 18), myelodysplastic syndrome (MDS; *n* = 6), severe aplastic anemia (SAA; *n* = 18), primary immunodeficiency (PID; *n* = 14), juvenile myelomonocytic leukemia (JMML; *n* = 3), neuroblastoma (NBL; *n* = 22), Ewing sarcoma (ES; *n* = 15), Hodgkin lymphoma (HD; *n* = 9), non-Hodgkin lymphoma (NHL; *n* = 3), germ cell tumor (*n* = 4), hemophagocytic lymphohistiocytosis (HLH; *n* = 2), or other diseases (*n* = 18) (Table 1).

### Incidence of MDR gastrointestinal bacteria

#### ***Overall***

The incidence of MDR GTI in all group was 44% (*n* = 77/175 cases). In allo-HCT, it was 56% (*n* = 66/117 cases) and among autologous transplantation recipients 19% (11/58 patients). The most common bacteria species were *Clostridium difficile* (CDI), found in 18% (31/175 cases), *Klebsiella pneumoniae* ESBL positive 7.4% (*n* = 13/175 cases), *E. coli* ESBL positive 4% (*n* = 7/175 cases), and *Enterococcus* HLAR 4.6% (*n* = 8/77 cases). In allogeneic setting, incidence of *Klebsiella pneumoniae* ESBL positive was 10% (*n* = 12/117 cases), *E. coli* ESBL positive 7% (*n* = 8/117 cases), and CDI accounted for about 22%. Detailed information concerning strains in GTI are presented in Table 2.

**Table 2:** Characteristics of bacterial MDR strains in GTI2

Bacteria	Total 77 (100%)
*Clostridium difficile*	31 (40.3%)
*Klebsiella pneumoniae* ESBL	13 (16.9%)
*E. coli* ESBL	7 (9.1%)
*Enterococcus faecium* HLAR	8 (10.4%)
*Enterobacter cloacae* ESBL	4 (5.2%)
*Klebsiella pneumoniae* KPC	2 (2.6%)
*Pseudomonas aeruginosa* MBL	2 (2.6%)
*Enterobacter absurdiae* AmpC\	2 (2.6%)
*Enterococcus faecium* VRE	2 (2.6%)
*Enterobacter aerogenes* AmpC	1 (1.3%)
*Morganella morgani ESBL*	1 (1.3%)
*Serratia marcescens* AmpC	1 (1.3%)
*Acinetobacter baumannii* MBL	1 (1.3%)
*Citrobacter freundii* ESBL	2 (2.6%)

Legend. ESBL, extended-spectrum β-lactamase; VRE, vancomycin-resistant *Enterococcus*; MBL, metallo-beta-lactamase; HLAR, high-level aminoglycoside-resistant, KPC, *Klebsiella pneumoniae* carbapenemase

#### ***GTI1 Group***

Ninety-eight patients (56%) including 59 boys and 39 girls did not develop signs of MDR GTI. Median age was 7.1 years (range 0.4–23.5 years). Almost half of patients (47/98) underwent autologous transplantation. Patient distribution for diagnosis, donor, stem cell source, conditioning regimen, and GVHD are shown in Table 3.

**Table 3:** Incidence of GTI

Diagnosis	GTI2	GTI1
Total	77 (100%)	98 (100%)
ALL	18 (23.4%)	25 (25.5%)
AML	16 (20.8%)	2 (2.0%)
SAA/FA	11 (14.3%)	7 (7.1%)
MDS	5 (6.5%)	1 (1.0%)
JMML	2 (2.6%)	1 (1.0%)
HLH	2 (2.6%)	-
NHL	3 (3.9%)	-
HD	1 (1.3%)	8 (8.2%)
PID	2 (2.6%)	12 (12.2%)
NBL	2 (2.6%)	20 (20.4%)
ES	2 (2.6%)	13 (13.3%)
GERM	2 (2.6%)	2 (2.0%)
other	11 (14.3%)	7 (7.1%)
Neutrophil recovery		
Day < 15 after HCT	31 (40.3%)	52 (53.1%)
Day ≥ 15 after HCT	46 (59.7%)	46 (46.9%)
Graft Source		
CB	1 (1.3%)	-
BM	25 (32.5%)	35 (35.7%)
PB	51 (66.2%)	63 (64.3%)
Donor		
MUD	46 (59.7%)	27 (27.6%)
MSD	11 (14.3%)	21 (21.4%)
MMUD	5 (6.5%)	2 (2.0%)
HAPLO	4 (5.2%)	1 (1.0%)
AUTO	11 (14.3%)	47 (48%)
Conditioning regimen		
None	2 (2.6%)	1 (1.0%)
TBI-VP/TBI-VP-ATG	8 (10.4%)	12 (12.2%)
FLU-TREO-TT	34 (44.2%)	15 (15.3%)
BU-FLU-TT	-	2 (2%)
BU-CY-MEL	5 (6.5%)	1 (1%)
CY-FLU	19 (24.7%)	1 (1%)
TREO-MEL	3 (3.9%)	10 (10.2%)
BU-MEL	3 (3.9%)	17 (17.3%)
TREO-FLU	1 (1.3%)	6 (6.1%)
others	2 (2.6%)	33 (33.7%)
GVHD before GTI Total	43/77 (55.8%)	12/98 (12.2%)
Chronic	12 (27.9%)	1 (8%)
Acute	28 (65.11%)	11 (92%)
Acute + chronic	3 (6.9%)	-
n.a	11 (14.2%)	47 (48%)
Local. GVHD		
Skin	19 (44.2%)	11 (92%)
Gut ± skin	18 (41.8%)	1 (8%)
Liver ± others	6( 14%)	-
GVHD grade		
1	5 (11.5%)	4 (33.4%)
2	12 (28%)	6 (50%)
3	14 (32.5%)	2 (16.6%)
4	12 (28%)	-

Legend. TBI-VP, total body irradiation, etoposide; TREO-FLU-TT, treosulfan-fludarabine-thiotepa; BU-FLU-TT, busulfan-fludarabine-thiotepa; BU-CY-MEL, busulfan-cyclophosphamide-melphalan; ALL, acute lymphoblastic leukemia; AML, acute myeloid leukemia; SAA, severe aplastic anemia; FA, Fanconi anemia; MDS, myelodysplastic syndromes; HLH, hemophagocytic lymphohistiocytosis; JMML, juvenile myelomonocytic leukemia; NHL, non-Hodgkin lymphoma; HD, Hodgkin lymphoma; NBL, neuroblastoma; ES, Ewing sarcoma; GERM, germ cell tumor; PID, primary immunodeficiencies; MSD, matched sibling donor; CB, cord blood; MUD, matched unrelated donor; PB, peripheral blood; BM, bone marrow; MMUD, mismatched unrelated donor; GVHD, graft-versus-host disease

#### ***GTI2 Group***

Among children who developed symptoms (*n* = 77), there were 28 girls and 49 boys, at median age 7.0 years (range 0.3–18.1). Majority of patients 86% (*n* = 66 cases) underwent allogeneic-HCT, transplanted due to: acute lymphoblastic leukemia (ALL; *n* = 18/66), acute myeloblastic leukemia (AML; *n* = 16/66), myelodysplastic syndromes (MDS; *n* = 5/66), severe aplastic anemia (SAA; *n* = 11/66), primary immunodeficiency (PID; *n* = 2/66), juvenile myelomonocytic leukemia (JMML; *n* = 2/66).

Patient distribution for other diagnosis is presented in Table 3.

Majority of infected patients underwent myeloablative conditioning. In allogeneic setting, 46/66 patients underwent matched unrelated donor (MUD-HCT), 11/66 pts matched sibling donor (MFD-HCT), 5/66 pts mismatched unrelated donor (MMUD-HCT), and four patient was given a haploidentical peripheral blood stem cell transplantation. Bone marrow (BM) and peripheral blood (PBSCT) were transplant source in 25/77 and 51/77 cases, respectively. In one case, cord blood transplantation (CBT) was performed. Forty-three (55.8%) patients with GTI developed GVHD. Twenty-eight of them (65.1%) suffered from acute GVHD (aGVHD), 12 patients (27.9%) developed chronic GVHD, and three of them acute/chronic GVHD. Detailed information on GVHD grade and location are presented in Table 3.

Among allogeneic positive group, 26/66 patients (39.4%) developed *Clostridium difficile* (CDI) infection,12/66 patients (18.2%) had *Klebsiella pneumoniae* ESBL positive infection, 8/66 children (12%) suffered from *E. coli* ESBL positive and the same 6/66 patients (9%) developed *Enterococcus* HLAR infection. In two cases *Enetrobacter cloacae* ESBL positive was found in stool culture.

In the autologous GTI positive group, similarly to allogeneic, the most common were CDI infection accounted for about 45.5% (*n* = 5/11 patients). Two cases of *Enterobacter cloacae* ESBL positive and *Enetrococcus* HLAR were found in this group. *Klebsiella pneumoniae* ESBL positive and *E. coli* ESBL positive were diagnosed in single cases. There were no typical species related to allogeneic or autologous cohort. Detailed information concerning bacteria species are presented in Table 2.

#### Clinical course/manifestation of MDR GTI

Overall, 77 patients (44%) developed signs and symptoms of GTI: diarrhea (*n* = 40 cases), vomiting (*n* = 22 cases), fever (*n* = 55 cases), abdominal pain (*n* = 21 cases), and typhlitis (*n* = 6 cases). There were 5 patients who developed septic shock symptoms including one with megacolon toxicum. The median time to develop GTI infection was 22 days (range 0–318 days) from the beginning of conditioning regimen.

#### ***Treatment***

Patients were treated with broad-spectrum antibiotics. The mean period of treatment was 30 days (range: 5–210 days). Antibacterial treatment caused resolution of symptoms in majority of cases.

Treatment of CDI was successful in first-line therapy with metronidazole (*n* = 13) and vancomycin (*n* = 18). There were no children who required second drug: vancomycin or metronidazole. Probiotics were used rarely.

#### ***Deaths***

Overall, 16 patients died, including 11, who had experienced GTI; however, no death was attributed to gastrointestinal infection directly. In 6/16 cases, the death was secondary to generalized inflammatory response syndrome with multi-organ failure (MOF) caused by other bacterial infections or viral or fungal. The primary cause of other deaths was progression of underling disease (*n* = 4), GVHD (*n* = 2), accident (*n* = 1), CNS complications (*n* = 1), and others (*n* = 2).

### Multivariate analysis for risk factors for GTI incidence

In multivariate analysis, significantly higher incidence of GTI was found in children after MUD transplant (OR 3.09; 95% CI 1.29–7.43; *p* = 0.011). A lower incidence of GTI was observed in case of full match donor (OR 0.24; 95% CI 0.11–0.53; *p* = 0.001). Patient with GVHD before infection (OR 3.46; 95% CI 1.50–8.02; *p* = 0.004), had higher incidence of GTI. There was no significant impact of neutrophil engraftment, age, bone marrow source, and TBI on incidence of gastrointestinal infection, although there was a trend towards higher incidence of GTI in patients where neutrophil recovery was longer than 15 days (Table 4). Among children with CDI infection, significantly lower incidence of *Clostridium difficile* infection was found in children with full matched donor (OR 0.13; 95% CI 0.04–0.38; *p* = 0.0001), presence of acute GVHD (OR 0.22; 95% CI 0.07–0.64; *p* = 0.005) and gut GVHD (OR 0.02; 95% CI 0.01–0.23; *p* = 0.001) (Table 5). No significant impact of type of transplant (MUD vs MSD), age, type of conditioning, or neutrophil engraftment on CDI incidence was found.

**Table 4:** Multivariate logistic regression analysis of risk factors for GTI

Risk factors	OR (95%CI)	*p* value
Age > 5 years	0.96 (0.52–1.77)	0.911
TBI-VP vs other conditioning	1.20 (0.46–3.10)	0.702
Donor: MUD vs MSD	3.0 (1.29–7.43)	0.011
HLA match	0.24 (0.11–0.53)	0.001
PB vs BM	1.15 (0.61–2.17)	0.653
Neutrophil recovery > 15 day	0.59 (0.32–1.09)	0.09
GVHD before GTI infection	3.4 (1.50–8.02)	0.004

Legend. TBI-VP, total body irradiation-etoposide; MSD, matched sibling donor; MUD, matched unrelated donor; PB, peripheral blood; BM, bone marrow; GVHD, graft-versus-host disease

**Table 5:** Multivariate logistic regression analysis of risk factors for CDI

Risk factors	OR (95%CI)	*p* value
Age > 5 years	0.72 (0.31–1.65)	0.44
TBI-VP vs other conditioning	1.44 (0.38–5.45)	0.59
Donor: MUD vs MSD	1.85 (0.67–5.05)	0.22
HLA match	0.13 (0.04–0.38)	0.0001
Neutrophil recovery > 15 day	0.83 (0.37–1.83)	0.64
aGVHD before CDI	0.22 (0.07–0.64)	0.005
gut GVHD	0.02 (0.03–0.23)	0.001

Legend. TBI-VP, total body irradiation-etoposide; MSD, matched sibling donor; MUD, matched unrelated donor; PB, peripheral blood; BM, bone marrow; GVHD, graft-versus-host disease

## Discussion

We analyzed incidence and the clinical risk factors for MDR GTI after allo-HCT and auto-HCT in pediatric population. Incidence was 44% among enrolled patients. It was lower among autologous recipients (19%) than in allogeneic setting (56%). According to many authors, the risk of infection is higher in patients after allo-HCT than auto-HCT [8–10].

The most common bacteria species were *Clostridium difficile* (CDI), found in approximately 18% overall, 22% of allogeneic transplantation and 8.6% of autologous transplant. These results are in line with Boyle et al. who found that 11% of adults and 17% of children developed episode of CDI by day + 100 [11]. The incidence of CDI in HCT pediatric group varies between 2 and 27% according to some authors [12, 13]. When we compare it with our previous study [7], describing patients from years 2012 to 2015, the incidence in allogeneic and autologous setting is much higher nowadays (8.9% vs 22% and 6.7% vs 8.6%, respectively).

Looking for MDR Gram-negative bacteria incidence in our study, it was 26% overall. Balletto and Mikulska [14] showed similar incidence, highlighting that Enterobacteriaceae are the most frequent pathogens causing approximately 25% of BSI followed by pneumonia and gastrointestinal infections. In contrast Patriarca et al. presented in their study lower GN incidence at about 10% overall and 18% in allogeneic setting [15]. Incidence of *Klebsiella pneumoniae* ESBL positive in our study was 7.4%, *E. coli* ESBL positive was 4% overall, in allogeneic transplantation it was approximately 10% and 5%. In majority of the European countries, over 10% of all invasive infections caused by *E. coli* in 2012 were due to strains unsusceptible to 3rd-generation cephalosporins and the prevalence of ESBL producing strains in patients with hematological malignancies varies from 13% in Spain to 48% in Japan [16, 17].

Apart from *Clostridium difficile* and GN multidrug-resistant bacteria, we observed enterococcal infections quite often. Incidence of *Enterococcus* HLAR was 4% (*n* = 7/175 cases), and vancomycin-resistant Enterococcus (VRE) was 2% (*n* = 3/175 cases). According to Shono et al., in patient after allo-HSCT, simultaneous use of prophylactic antibiotics and antibiotic treatment of febrile neutropenia (FUO) leads to microbial diversity, which increases susceptibility to infections [3]. Whereas a healthy individual usually carries on the order of thousand different species, after allo-HCT, near dominance of the intestinal flora, particularly by a single species of Proteobacteria, Enterococcus or Streptococcus, is frequently observed. In particular exposure to the metronidazole and vancomycin is associated with Enterococcus domination. In accordance with the general European data [18, 19], there is a low incidence of VRE in European hematological centers with less than 5% of enterococci, being VRE in 67% according to the ECIL-4 questionnaire. In our study, incidence was much lower—VRE accounted for 20% of all enterococcal infections—but our data concerned only pediatric population.

Donor type seems to be an important risk factor causing infections after HCT including GTI. Styczyński et al. highlighted that the risk of infections after HSCT depends on the type of transplantation. The low-risk group is concerning autologous HSCT; moderate-risk, matched sibling donor HSCT with no GVHD; and high-risk, unrelated, mismatched, haploidentical, cord blood HSCT [8]. Similarly, in our study in multivariate analysis, incidence of GTI was higher in matched unrelated donor transplantation (MUD) than in children transplanted from siblings. Other studies have presented that they did not observe any significant correlation between donor type and occurrence of MDR infections [15].

Full HLA matching was connected with lower incidence of gastrointestinal tract infections in our analysis. Similarly, among children with CDI, significantly lower incidence of *Clostridium difficile* infection was found in children with full matched donor. It was not in line with other studies, where no significant impact was found for HLA match and infection incidence [20].

The connection between gastrointestinal infections and GVHD has been studied for many years. Majority of authors observed that risk of infections is higher in patients after allogeneic transplantation and in those with GVHD [8–10, 14].These observation are in line with results of our uni-analysis, where patient with GVHD before infection, had higher incidence of GTI overall. In a CDI group, we did not observe such correlation. Significantly lower incidence of *Clostridium difficile* infection was found in children with presence of acute GVHD and gut GVHD. More recent retrospective study of 15 pediatric patients presented by Waldrip et al. reported that the use of antibiotics effective against anaerobic bacteria has resulted in a significant increase in GVHD with a reduction of gut anti-inflammatory Clostridia. More over addition of clindamycin to levofloxacin in a mouse model of GVHD reduced Clostridia and exacerbated GVHD [21]. Similarly, other studies showed that Clostridiales may play important anti-inflammatory homeostatic roles, including upregulation of Treg cells through the production of the short-chain fatty acids (SCFA) butyrate, which increased the recovery of intestinal epithelial cells (IEC) damage after allo-HSCT, and the re-introduction of a mixture of 17 strains of human Clostridiales isolates, which produce high levels of butyrate, prolonged the survival of mice with GVHD. Clinical data indicate that increased abundance of the Blautia genus, which belongs to the class Clostridia, is significantly associated with less GVHD-related mortality and improved overall survival [22–27].

There was no significant impact of neutrophil engraftment on incidence of gastrointestinal infection in our study, although there was a trend towards higher incidence of GTI in patients whose neutrophil recovery was longer than 15 days. It was in line with other studies, where incidence and severity of infections after HCT strongly correlated with time of neutrophil engraftment [9, 10, 15].

We did not find significant impact of bone marrow source, TBI on incidence of gastrointestinal infection. In contrary to our analysis, Patriarca et al. observed that such factors as conditioning regimen, stem cell sources were significantly associated with development of infections caused by GN MRD bacteria [15]. Conditioning regimen with TBI was an important risk factor for developing CDI too in other studies [13, 28, 29]. Presented authors have analyzed adult or mixed (adult and/or children) population, so it could influence results. In other pediatric analysis concerning CDI in children after HCT, the authors did not find conditioning regiment, and TBI is a significant factor for CDI [7].

The main symptoms of GTI in our study were diarrhea, vomiting, fever, abdominal pains, and syndromes of typhlitis. There were 5 patients who developed symptoms of septic shock and one with megacolon toxicum. According to Tuncer et al., infectious etiologies of diarrhea account for only 10–15% of cases, yet diarrhea at any time after transplant should always prompt obtaining stool studies for CDI as well as bacterial, parasitic, and viral cultures if indicated [30–32]. Lee et al. presented in their study that the major infectious complication of GI after HCT were typhlitis, and pseudomembranous enterocolitis [33]. The clinical manifestation included abdominal pain or tenderness, fever accompanied by neutropenia. Symptoms occurred mainly during 30 days after HCT. Megacolon toxicum was caused by motility problems related to CDI infection or mega-chemotherapy. It appeared during post-engraftment period (days 31–100 after HCT) [30, 34]. In our study, majority of typhlitis cases appeared between 20 and 60 days after HCT, one case of megacolon was diagnosed 90 days after transplant. The median time to develop all GTI infection in our study was 22 days (range 0–318 days) from the beginning of conditioning regimen.

The influence of gastrointestinal tract infection after HCT on survival is crucial. According to some studies, gastrointestinal system involvement is one of the main complications seen in the recipients of HCT, and it is also a major cause of morbidity and death [15, 34]. Balletto et al. highlighted that bacterial infections consisting from bloodstream infection followed by pneumonia and gastrointestinal infections are the major complications after HCT. Infections caused by Gram-negative rods used to be the main cause of infection-related mortality in patients with neutropenia [35]. In contrast, some authors think that, although MDR GN infections have been recognized as leading cause of mortality after solid organ transplantation, their epidemiology and impact on patients after HCT and with hematologic malignancies have been not enough studied [36–39]. In that study, the main cause of deaths was not attributed to GTI infection but rather to progression of underlying disease. We observed 16 deaths, including 11, who had experienced GTI; however, no death was attributed to MDR gastrointestinal infection directly. In 6/16 cases, the death was secondary to generalized inflammatory response syndrome and MOF, caused by other infections (bacterial, viral, or fungal). The primary cause of other deaths was progression of underling disease, CNS complications, and road accident.

Our study has several limitations. Firstly, there was no unification in approach to fluoroquinolone prophylaxis in children below 4 years of life which differed between centers and screening for GTI which could influence incidence of bacterial infections/colonization too. Second, we studied both (in one cohort) autologous and allogeneic transplant recipients who had different risk of infections and mortality. Another limitation was lack of appropriate unified measures used to classify patient as “GTI positive.” Inclusion was based on positive stool culture and presence of clinical symptoms, but it was subjective and done in a few centers.

In conclusion, MDR GTI remain frequent and troublesome particularly in clinically severe stage, often causing longer hospitalization but rarely contributed to death among children after HCT. GTI occurred in 44% of enrolled children. We have identified different risk factors in HCT recipients of developing MDR GTI, although there are limitations to this analysis. HLA matching is influencing predisposition to develop MDR GTI. Patients transplanted from full HLA match donor have lower chance to develop GTI. Donor type seems to be an important risk factor causing infections after HCT including MDR GTI. Children transplanted from MUD are more predisposed to develop GTI infection. Acute GVHD has been shown to be an important risk factor for developing GTI, but only among children with GN MDR infections. Children having CDI are at the lowest risk of GVHD, what is connected with beneficial role of Clostridiales and production of SCFA butyrate. MRD GTI rarely cause death, but very often breakthrough strains with non-resistant bacteria can be fatal (and responsible for more than 30% of deaths in these population).

## Supplementary Information

Below is the link to the electronic supplementary material.

Supplementary file1 (PPTX 37 KB)

## Data Availability

The data that support the findings of this study are available on request from the corresponding author. The data are not publicly available due to information that could compromise the privacy of research participants.
