# Supplementary material for: Analysis of incidence and risk factors of the multidrug resistant gastrointestinal tract infection in children and adolescents undergoing allogeneic and autologous hematopoietic cell transplantation: a nationwide study
Source: Ann Hematol. 2021 Oct 21;101(1):191–201. doi: 10.1007/s00277-021-04681-y (PMC8720737; doi:10.1007/s00277-021-04681-y)
Supplement: Supplementary file 1 — Supplementary file1 (PPTX 37 KB) [file 277_2021_4681_MOESM1_ESM.pptx]

## Slide 1
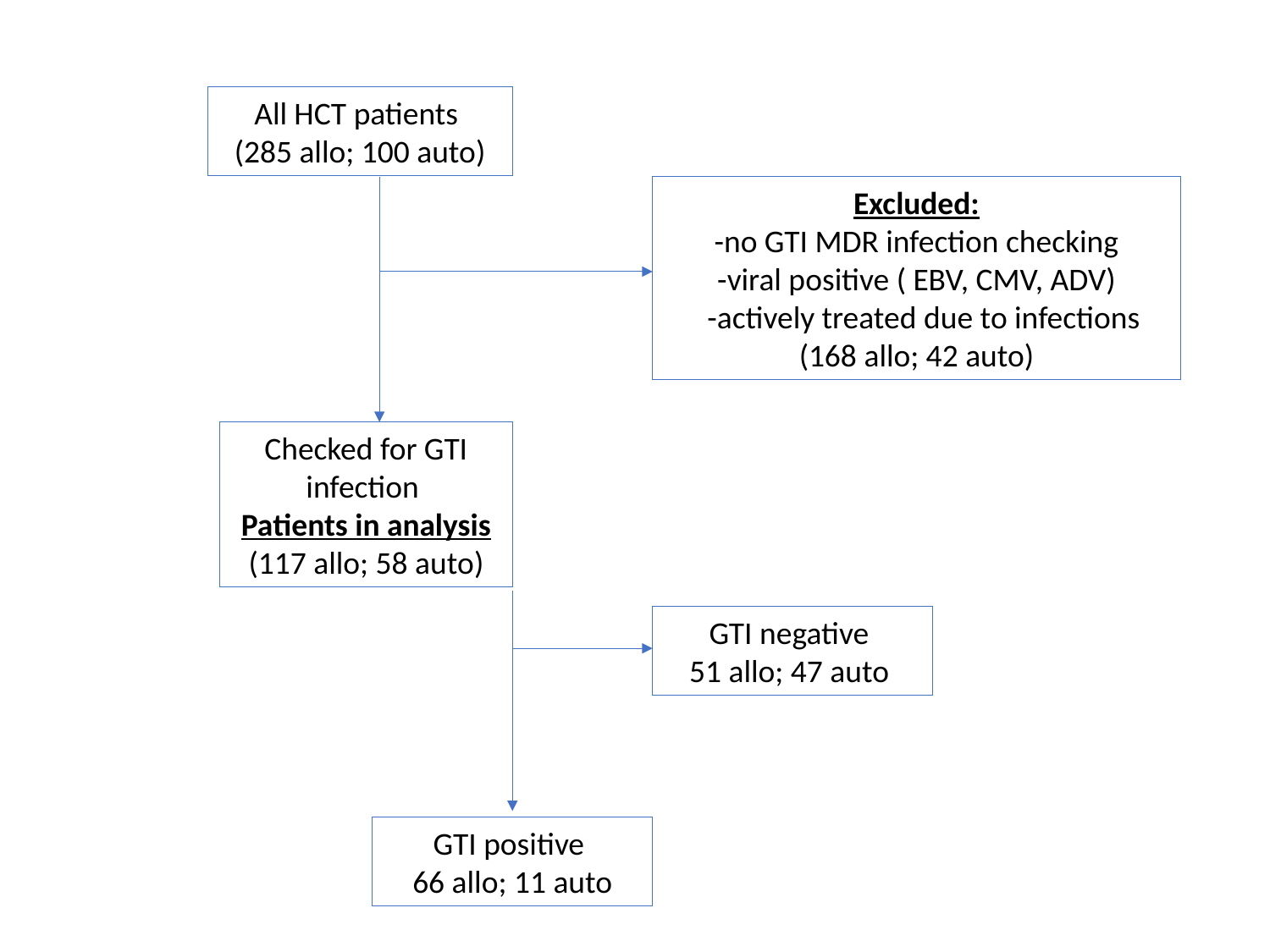

All HCT patients
(285 allo; 100 auto)
Excluded:
-no GTI MDR infection checking
-viral positive ( EBV, CMV, ADV)
 -actively treated due to infections
(168 allo; 42 auto)
Checked for GTI infection
Patients in analysis
(117 allo; 58 auto)
GTI negative
51 allo; 47 auto
GTI positive
66 allo; 11 auto
